# Demographics and donor motivation drive declining blood donations: A 15‐year study in Germany reflecting trends in high‐income countries

**DOI:** 10.1111/vox.70194

**Published:** 2026-02-06

**Authors:** Sophia Oesterreicher, Kerstin Weitmann, Antje Sieg, Thomas Thiele, Kirstin Stüpmann, Doris Gloger, Wolfgang Hoffmann, Andreas Greinacher, Linda Schönborn

**Affiliations:** ^1^ Institut für Transfusionsmedizin Universitätsmedizin Greifswald Greifswald Germany; ^2^ Universitätsklinik für Transfusionsmedizin und Zelltherapie, Medizinische Universität Wien Wien Austria; ^3^ Institut für Community Medicine Universitätsmedizin Greifswald Greifswald Germany; ^4^ Institut für Transfusionsmedizin Universitätsmedizin Rostock Rostock Germany; ^5^ DRK‐Blutspendedienst Mecklenburg‐Vorpommern Rostock Germany; ^6^ Haema AG, Blutspendezentrum Rostock Rostock Germany

**Keywords:** blood donations, blood supply, demographic ageing

## Abstract

**Background and Objectives:**

Ageing populations in high‐income countries reduce the proportion of potential blood donors while increasing transfusion demand. Sustaining an adequate blood supply requires higher donor motivation among younger age groups. We analysed long‐term trends in whole blood donations (WBDs) in one German federal state as an indicator for challenges that may arise in other high‐income countries.

**Materials and Methods:**

In our prospective longitudinal study (starting 2005), we obtained the age and sex of the donors of all WBDs in the German federal state Mecklenburg‐Western Pomerania in 2019 and 2020 and compared them with the data from 2005 to 2015. Population data from the German statistical office were used to predict future WBD in two models, population‐based and population‐based with behaviour‐adjusted prediction.

**Results:**

WBD decreased from 118,419 in 2005 to 83,871 in 2019 (−29.2%) and 76,912 in 2020 (−35.1%). Donation rates per 1000 inhabitants declined by 19.1% between 2005 and 2019, indicating a loss of donor motivation beyond demographic effects. Based on the donation numbers of 2019, we predict a further decline of WBD in 2030 by −12.7% (population‐based projection) or −15.1% (behaviour‐adjusted projection), respectively.

**Conclusion:**

The decline in blood donations is no longer solely driven by demographic changes but also by reduced motivation among younger donors. As ageing populations and changing donor behaviour are common to many high‐income countries, these findings likely reflect an emerging international trend. Targeted strategies to recruit and retain young donors are urgently needed to ensure sustainable blood supplies in ageing societies.

**Highlights**

- Whole blood donations declined by 35% in 15 years, exceeding the predicted effects of the demographic change.
- Additionally, blood donation rates among younger age groups have markedly decreased.
- Similar trends may occur in other high‐income countries, potentially challenging future blood supply.

## INTRODUCTION

Health care systems of high‐income countries are challenged by an ageing population. In Germany, the federal states formerly belonging to the German Democratic Republic (Eastern Germany) are more affected by this demographic change than other parts of the country. The pronounced decline of birth rates after the German reunification in 1990 and the large number of young females who migrated to Western parts of Germany further skewed demography [1]. As a consequence, the effects of demographic changes become apparent about 10 years earlier in Eastern Germany than in the Western federal states [2].

The demographic change causes a decline in blood donation numbers, mainly by a shrinking population in the age groups <50 years, paralleled by an increasing transfusion demand in age groups >60 years as they grow in population size [3, 4, 5]. Since 2005, we perform one of the largest prospective longitudinal studies on blood donations and demand, enrolling all whole blood donors and red blood cell (RBC) concentrate recipients in the German federal state Mecklenburg‐Western Pomerania [4, 6, 7]. Until 2015, one of the main results of this study was that the number of whole blood donations (WBDs) mainly follows the demographic trend [4, 6, 7]. This is highly relevant in the context of demographic changes, as in Europe a large proportion of whole blood donors belong to the age groups >45 years [3, 5, 8, 9, 10]. Many of them will shift from the donor pool to the transfusion recipient side within the next years due to age‐related health issues.

In parallel to the demographic change, changes in medical practice (minimally invasive surgery, patient blood management, less cytotoxic tumour therapies, etc.) resulted in a substantial decrease of the transfusion demand [11]. Little is known about the balance between these two effects. This requires regular monitoring of blood donation numbers and transfusion demand to recognize the potential development of a shortfall in the blood supply. The aim of this 5‐years follow‐up of our prospective study is to analyse changes of the effect of the demographic change on the characteristics of whole blood donors and WBD rates for the years 2019 and 2020 in the German federal state of Mecklenburg‐Western Pomerania in comparison with the data obtained in the years 2005–2015. By adding this new observation period, the present dataset reveals developments that were not observed in earlier study periods, most notably the recent decline in donation rates per 1000 population among younger donors, which now occurs in addition to the demographic reduction in the size of this age group. At the same time, long‐standing patterns remain unchanged: donation activity continues to shift into older age groups as the baby‐boom generation is ageing, which has consistently formed a major contributor to overall donation numbers.

## MATERIALS AND METHODS

The data were provided by the four blood donation services operating in the federal state of Mecklenburg‐Western Pomerania (blood donation services of the University hospitals Rostock and Greifswald; German Red Cross blood donation service; and the Haema AG blood donation service [private blood donation service]). For each WBD in Mecklenburg‐Western Pomerania in the years 2005, 2010, 2015, 2019 and 2020, the following characteristics were determined: age (or date of birth), sex of the donor, donor identity number (ID) and date of blood donation.

To calculate the sex‐ and age‐specific numbers of donations per 1000 inhabitants, data of the Statistical Office of Mecklenburg‐Western Pomerania were used. The office provided data of the population in 1‐year age groups for females and males for 2005, 2010 (not shown), 2015, 2019 and 2020 [12].

Although we used an interval of 5 years for the analyses, due to the COVID‐19 pandemic we decided to additionally analyse the year 2019, one year before the outbreak of the pandemic and the regular examination year 2020, to avoid any misinterpretations of WBD numbers due to pandemic‐associated changes in blood donor behaviour. Population data were obtained from the Federal Statistical Office for the comparison of the actual and predicted demographic structure of Mecklenburg‐Western Pomerania [2, 13].

### Prediction of future blood supply

We used two complementary projection models to estimate future WBDs. For both models, 2019 was used as the reference year for observed donation rates, as it reflects blood donation behaviour unaffected by pandemic‐related restrictions.

#### Projection model A—Population‐based projection

The number of donations per age group in 2019 was first divided by the number of inhabitants in that age group to obtain age‐specific donation rates. In a second step, this rate was multiplied by the expected future population per age group provided by the Federal Statistical Office [2, 13]. The resulting projections are shown with a deviation of plus 5% and minus 5% around the expected value. This model is consistent with the approach used in our previous longitudinal analyses and describes the effect of the demographic change on future blood supply [4, 6, 7].

#### Projection model B—Behaviour‐adjusted projection

In order to incorporate the recently observed decline in donation rates into the model, we calculated the percentage change in donations per age group and population from 2015 to 2019. We multiplied this percentage by the number of donations projected for 2030 calculated in model A. Afterward, the curve was smoothed by calculating the average value from the sum of donations per age group and the number of donations from the preceding and following age groups.

The pseudonymization of the donor ID was performed by the independent Trusted Third‐Party of the Universitätsmedizin Greifswald. Only pseudonymized data were analysed.

The institutional ethics review board of the Universitätsmedizin Greifswald approved the study (BB 111/20). In the analyses, exclusively pseudonymized data were used.

## RESULTS

### Impact of the demographic change on the potential donor population

**FIGURE 1:**
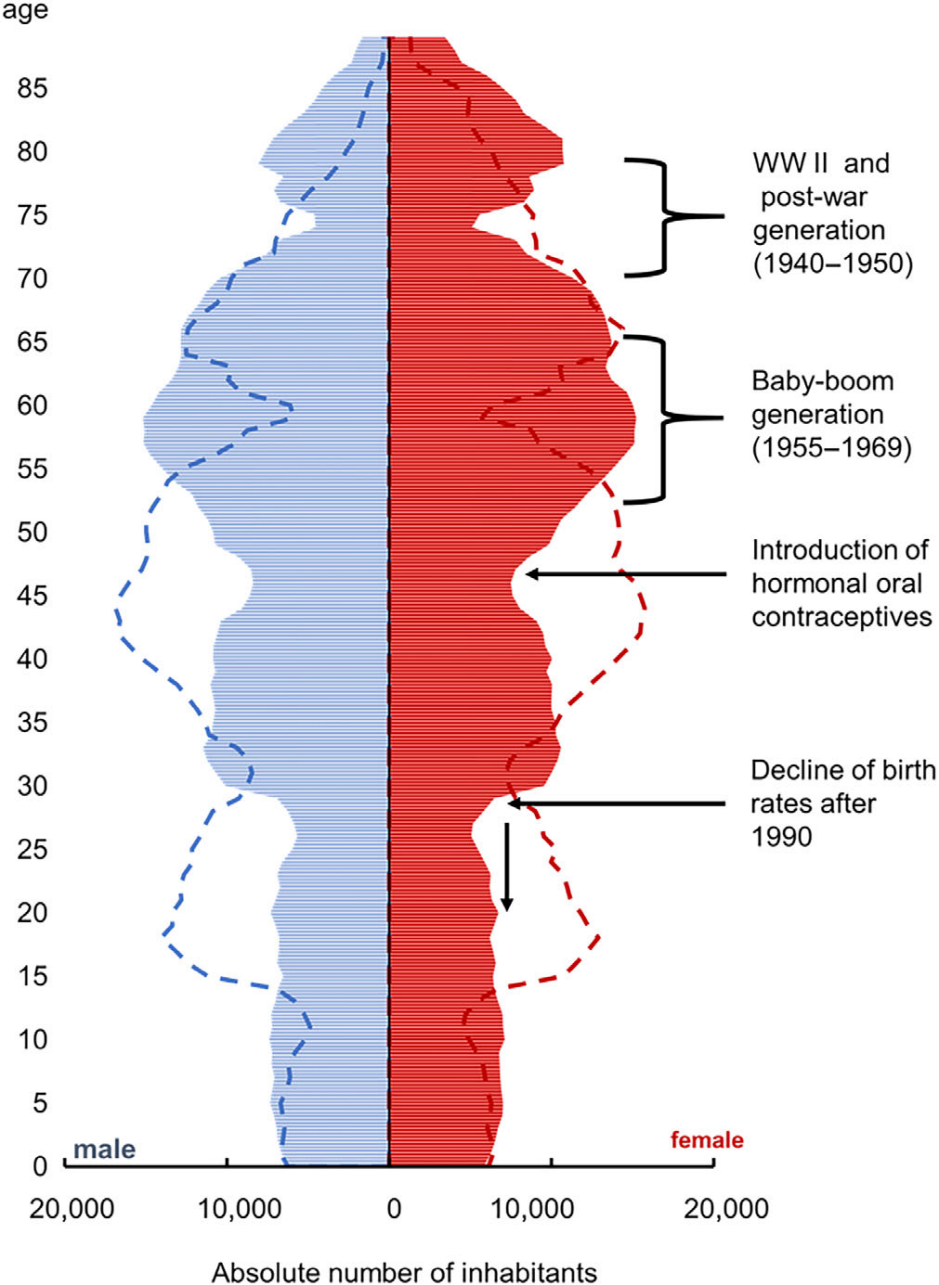
Population of Mecklenburg‐Western Pomerania (‐ ‐ ‐ 2005, 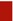 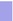 2020). Four major events had an impact on the demographic structure in the State: World War II (WW II), the baby‐boom generation (born 1955–1969), introduction of the hormonal oral contraceptives in the 1970s, and German reunification in 1990.

While from 2005 to 2020 the population of Mecklenburg‐Western Pomerania in the age groups <65 years decreased from 1,371,237 (80.3% of the population) to 1,194,612 inhabitants (74.2% of the population), the age groups ≥65 years increased from 336,029 (19.7%) to 416,162 inhabitants (25.8%) [12, 14]. Figure 1 shows the age structure of Mecklenburg‐Western Pomerania and its ageing population.

### Absolute number of WBDs

The trend of declining WBD numbers between 2005 and 2015 (−18.0%) further continued. Compared to 2015, within only 5 years, WBD numbers further declined by −13.6% to 83,871 WBD in 2019 and by −20.7% to 76,912 WBD in 2020 (total decline 2005–2020, −35.1%). A 35% decrease in blood donation numbers—while the population aged 18–69 years only decreased by 13.7%—indicates an additional loss of blood donors beyond what is expected by the demographic change.

### Age‐related differences in the development of WBD

**FIGURE 2:**
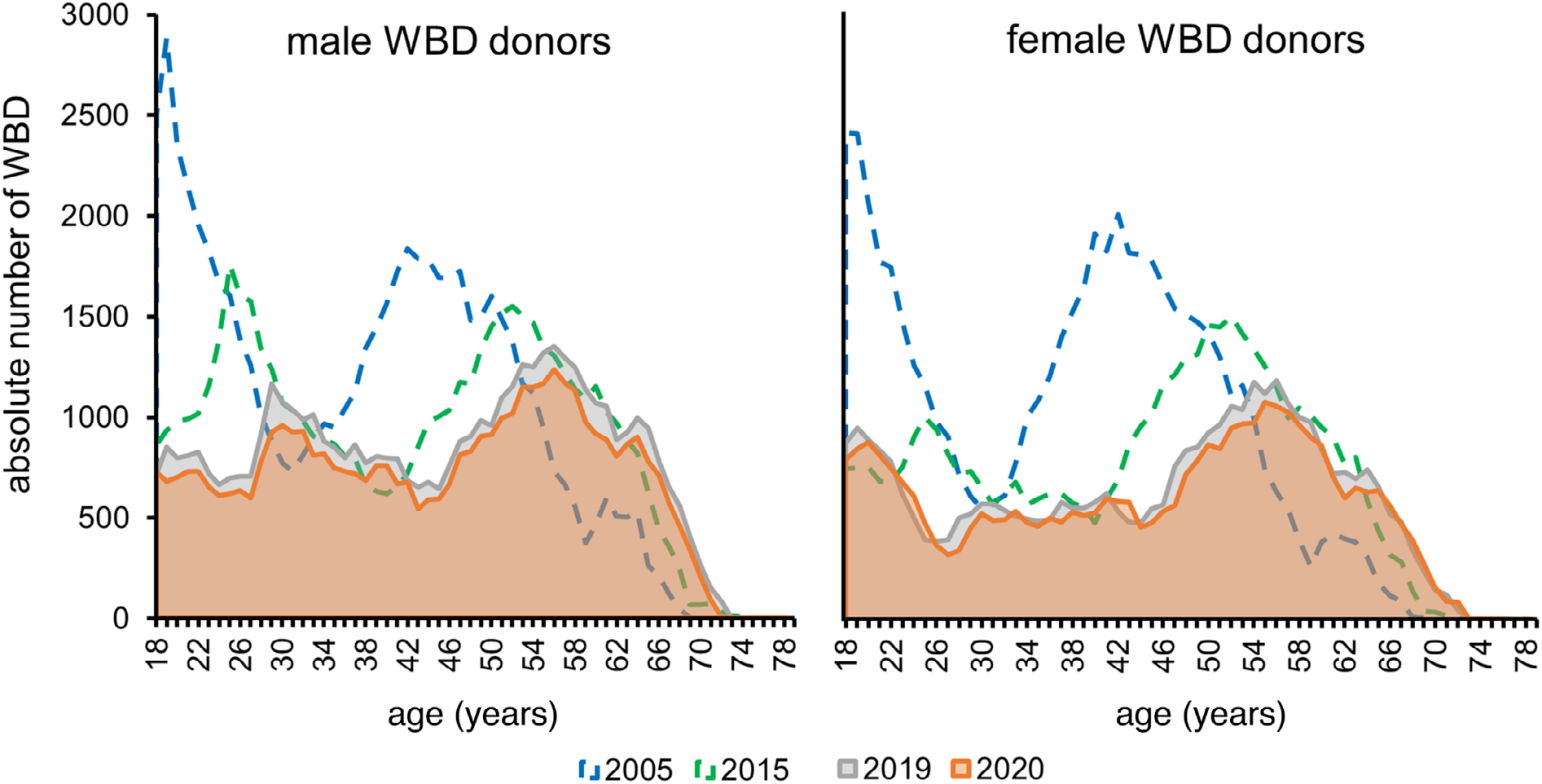
Absolute number of whole blood donations (WBD) by sex in 2005 (blue hatched line), 2015 (green hatched line), 2019 (grey solid line), and 2020 (orange solid line). Between 2005 and 2020, the decline in the absolute number of WBD is remarkable. The number of blood donors younger than 30 years substantially decreased. In addition, the second peak in 2005 donated by donors 35–50 years old shifted to the age group 50–65 years in 2020. This shift reflects the ageing of the baby‐boom generation. The peak resembling the largest blood donor group will most likely further decrease during the next 10–15 years due to the ageing of the population.

The decline of WBD is especially pronounced in the age group 18–30 years with −60.6% since 2005 (Figure 2; 39,199 WBD in 2005; 24,475 in 2015; 17,337 in 2019; 15,445 in 2020). In comparison, the number of WBD in the age group >30 years ‘only’ decreased by −22.4% since 2005 (79,220 WBD in 2005; 72,570 in 2015; 66,534 in 2019 and 61,467 in 2020).

The age group with the highest absolute number of WBD shifted from 40–<45 years in 2005 (18,184 WBD, 15.4% of overall WBD) to 50–<55 years in 2015 (14,745 WBD, 15.2%) and to 55–<60 years in 2019 (11,776 WBD, 14.0%) and 2020 (10,947 WBD, 14.2%) (Figure 2). This reflects the ageing of the baby‐boom generation (born 1955–1969). Nearly half of the WBD were donated by donors ≥50 years of age (45.2% in 2019 and 45.9% in 2020).

**FIGURE 3:**
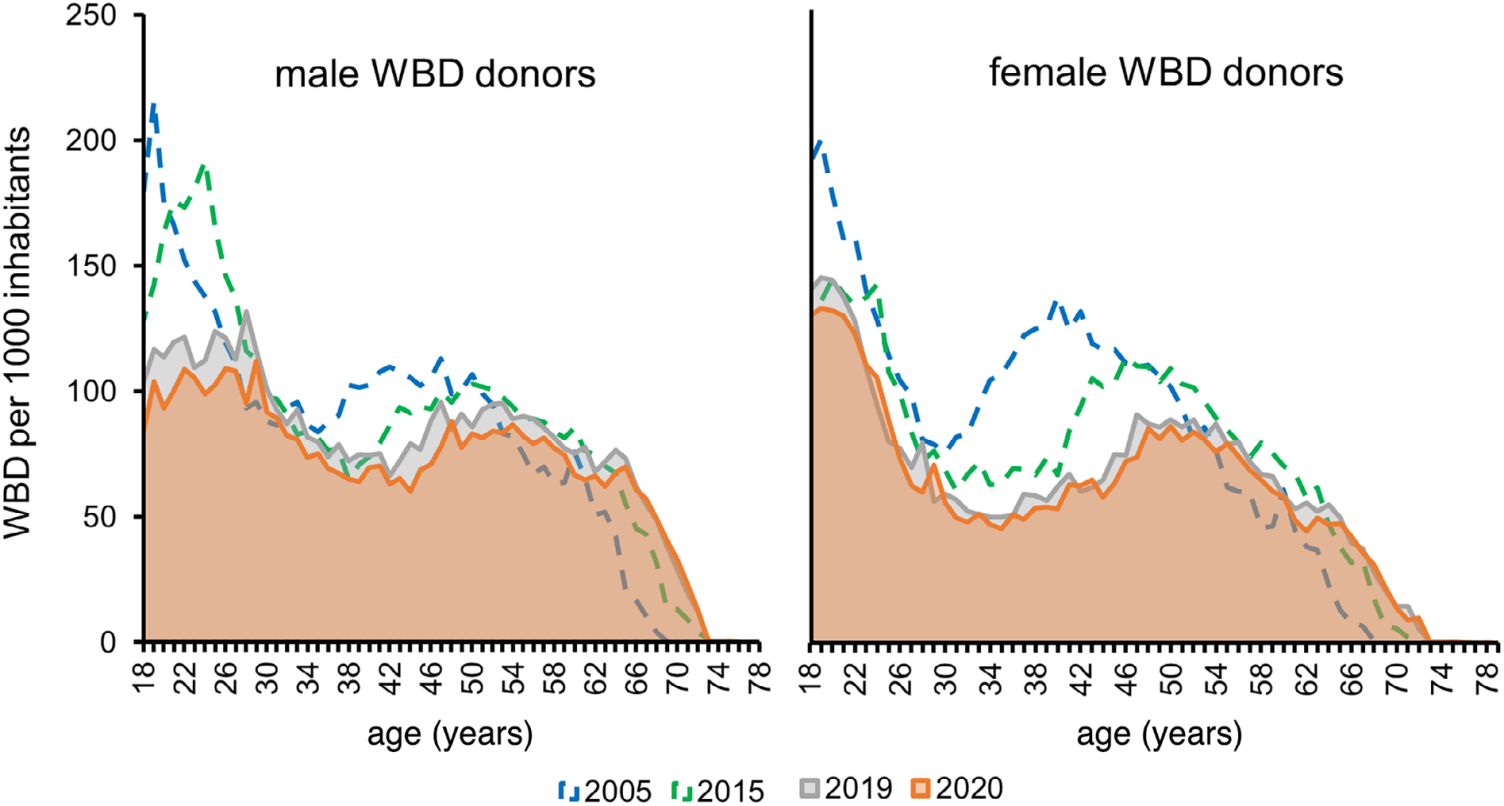
Whole blood donations (WBDs) per 1000 inhabitants by sex. The change in WBDs per 1000 inhabitants indicates a substantial decline in the motivation to donate blood. In the age groups 18–50 years, the orange solid line (donations/1000 population per age group) is substantially below the hatched blue (2005) and hatched green (2015) line. This is especially pronounced in men younger than 30 years and women in the age group 30–45 years. This decrease is alarming as it aggravates the decline in blood donation numbers due to the demographic change.

The donation rates per 1000 inhabitants per age group are shown in Figure 3. The donation rate per 1000 inhabitants over the age groups 18–69 years decreased by −19.1% from 95.1/1000 inhabitants in 2005 to 76.9/1000 inhabitants in 2019 and 70.6/1000 inhabitants in 2020 (−25.8%). This additional decrease in blood donation numbers per existing 1000 population by 26% is extremely worrisome as it substantially aggravates the effects of the demographic change.

### Sex‐related differences in the development of WBD

The proportion of WBD by women decreased from 48.0% in 2005 to 45.0% in 2015, 43.6% in 2019 and 44.7% in 2020. Consequently, the decline in WBD numbers was also more pronounced in female than in male blood donors. The number of WBD by women decreased by −39.5% from 2005 to 2020 (56,805 WBD in 2005; 43,637 in 2015 [−23.2%]; 36,596 in 2019 [−35.6%] and 34,372 in 2020 [−39.5%]). The number of WBD by men decreased by ‘only’ −31.0% from 2005 to 2020 (61,614 WBD in 2005; 53,402 in 2015 [−13.3%]; 47,275 in 2019 [−23.3%] and 42,540 in 2020 [−31.0%]). Only in the age group 18–<30 years was the decrease in WBD more pronounced in male than in female donors (2005–2020: −62.6% in males vs. −58.2% in females).

### Numbers of WBD per donor per year

**FIGURE 4:**
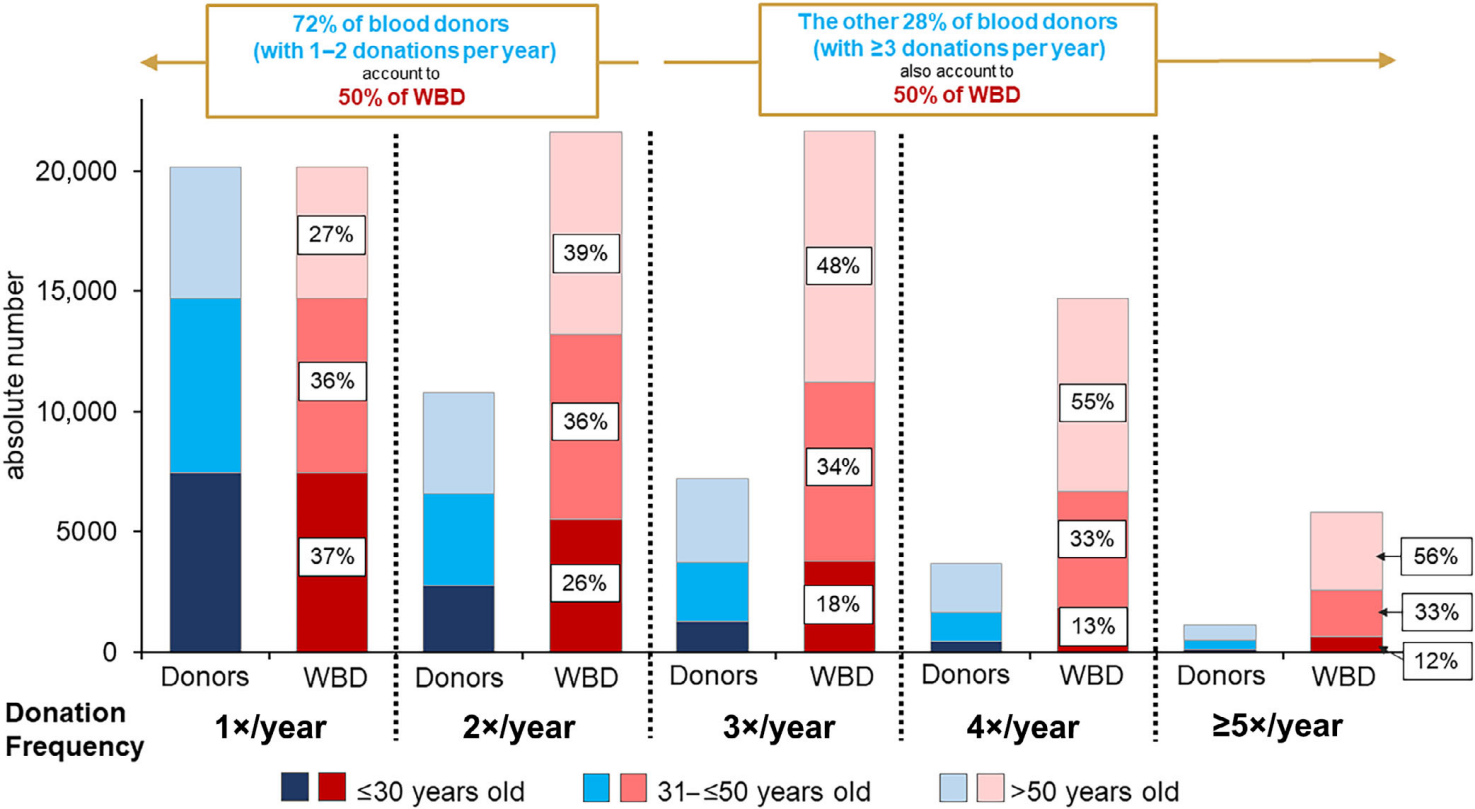
Absolute number of blood donors and whole blood donation (WBD) according to the donation frequency and donor age in 2019. The blue coloured bars show the absolute number of blood donors in the respective group, the red bars the resulting number of donated WBD. While the proportions of the age groups ≤30 years old and 31–≤50 years old are similarly distributed among those who donate once a year (37% and 36%, respectively), the proportion donated by donors >50 years old (27%) is smaller in this group. However, this ratio changes remarkably with increasing donation frequency: The more frequently blood is donated, the greater the proportion of older donors >50 years old and the lower the proportion of young donors ≤30 years old. This figure also visualizes the importance of reliable repeat donors for the overall blood supply: Those who donate one to two WBD per year (about 72.0% of all blood donors) account for about 50% of all WBDs. The other 28.0% of blood donors, who donate ≥3 times per year, also account for about 50% of all WBDs. In this group of donors donating ≥3 times per year, 50.8% of donors are 50 years or older, emphasizing the large impact of the successive retirement of the baby‐boom generation from the donor population on the overall blood supply over the next 10–15 years.

In 2019, 42,941 blood donors donated 83,871 WBD (mean donation frequency of 1.95 WBD/blood donor; 1.91 WBD/blood donor in 2020). The donation frequency depending on the donors' age groups is shown in Figure 4. In the group that donates 1 WBD per year, the age groups of ≤30 years and 31–≤50 years are equally represented (37% and 36%, respectively), whereas the donors >50 years old donate 27% of the overall WBD. However, as the donation frequency increases, the proportion of ≤30 year old donors decreases and the proportion of >50 year old donors increases steadily. Among those blood donors who donate four times or more WBD, the donors older than 50 years account for more than half of all WBD, whereas the proportion of ≤30 years old donors falls far below 15%.

Importantly, those who donate one to two WBD per year (about 72.0% of all blood donors) account for about 50% of all WBDs. The other 28.0% of blood donors, who donate ≥3 times per year, also account for about 50% of all WBDs. This highlights how important the few, but reliable repeat donors are for the overall blood supply. The fact that 50.8% of these repeat donors (≥3 WBD per year) are 50 years or older emphasizes that the successive retirement of the baby‐boom generation from the donor population over the next 10–15 years will have a particularly large impact on the overall blood supply.

### Differences between 2019 and 2020

The years 2019 and 2020 were compared in order to consider the effects of the COVID‐19 pandemic. The absolute number of WBD decreased by −8.3% from 83,871 WBD in 2019 to 76,912 WBD in 2020 (−6.1% by female donors, −10.0% by male donors). This difference is most likely an effect of the pandemic, as the age distribution of donors in these 2 years remained almost identical. In both years, approximately 46% of all WBD were donated by donors >50 years.

### Predicting the future blood supply

In our previous analyses, we showed that predicting the number of future WBD is possible under the assumption that age‐specific donation rates remain constant [4].

Based on the blood donation behaviour in 2015, we predicted a total number of 88,035 WBD for 2020 [4]. In 2019, the absolute number of WBD was 83,871 WBD (2019 was chosen as comparative value to exclude the effects of the pandemic on donation rates in 2020), which leads to a deviation between actual and predicted WBD of −4.7%.

**FIGURE 5:**
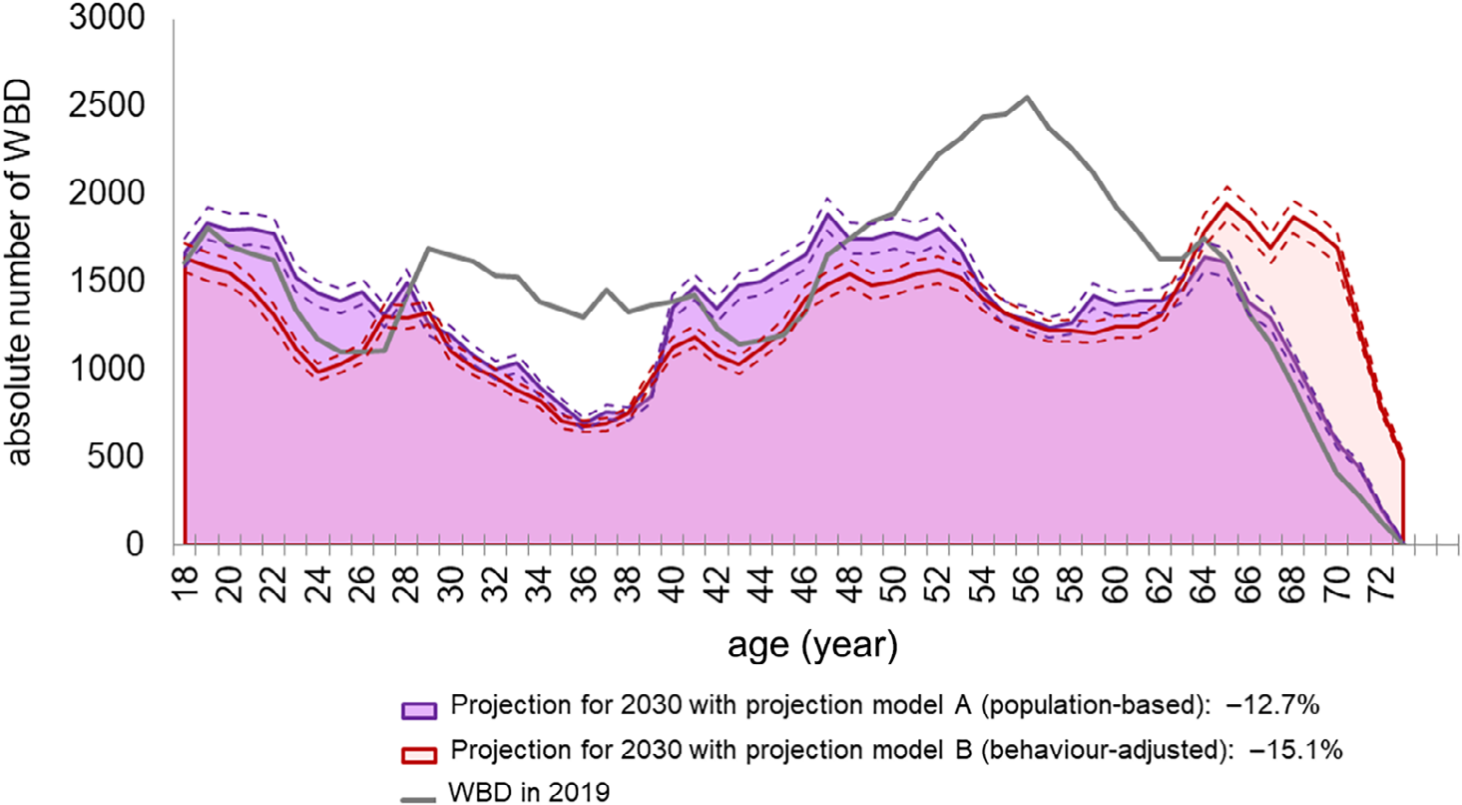
Projected numbers of whole blood donation (WBD) in 2030 based on two prediction models. The observed number of WBDs in 2019 is shown as a grey line. On the basis of the age‐ and sex‐specific donation rates observed in 2019, the projected number of WBD in 2030 was calculated using two complementary approaches. Projection model A (population‐based projection; purple shaded area) shows the predicted number of WBD in 2030 assuming that age‐ and sex‐specific donation rates remain constant at the 2019 level. Age‐ and sex‐specific population projections for 2030 were obtained from the Federal Statistical Office. Model A describes the effect of demographic change on future blood supply. Projection model B (behaviour‐adjusted projection; red shaded area) builds on model A but additionally incorporates the recently observed decline in age‐specific donation rates between 2015 and 2019, thereby capturing both demographic and behavioural changes in WBD (for detailed calculation see Materials and Methods section). In total, projection model A estimates 73,198 WBD for 2030, while projection model B predicts 71,172 WBD, reflecting an additional decline of 2026 WBD (−2.7%) when recent behavioural changes will continue. For both models, the dashed lines indicate a ±5% range around the respective projection to illustrate the potential variation in donation numbers.

For the projection of the blood supply in 2030, we applied two complementary projection models (Figure 5). Projection model A (population‐based projection) assumes constant age‐specific donation rates as observed in 2019 and yields an estimated 73,198 WBD for 2030, representing a further decline of −12.7% compared to 2019.

In contrast, projection model B (behaviour‐adjusted projection) incorporates the recently observed decline in donation rates observed between 2015 and 2019. After applying the age‐specific percentage changes, this model predicts 71,172 WBD for 2030, which is 2026 WBD fewer (−2.7%) than predicted by model A (accounting to a decline of 15.1% of WBD numbers compared to 2019). Notably, in prediction model B, the reduction in donation numbers is not uniform across age groups. While most younger age groups contribute substantially to the overall decline, the projection indicates a partial compensatory increase among donors aged >65 years (Figure 5, light‐red shaded area), reflecting both demographic growth in this age group and the comparatively stable or slightly rising donation rates observed between 2015 and 2019.

This illustrates the additional impact of behavioural changes on future blood supply beyond demographic effects alone.

## DISCUSSION

Mecklenburg‐Western Pomerania has been proven over many years as a model region to analyse the impact of demographic changes on blood donation rates and transfusion demand. The current analysis including the years 2019 and 2020 corroborates the robustness of our prediction model for the number of WBD with an error of about 5%. Importantly, our prediction model overestimated the number of WBD in the past. A highly relevant result of the new 5‐year observation period 2015–2019/2020 is that we observed a further substantial decline in WBD beyond what has been expected based on the demographic change. This indicates a decrease in the willingness to donate blood. This loss of motivation to donate blood is most pronounced in the next generation of blood donors, in the age group of 18–30 years (Figure 3). This requires major activities of the transfusion medicine community to reach these population groups and to motivate them to become blood donors.

The baby‐boom generation (born 1955–1969) remains the group with the highest number of WBD. As shown in Figure 2, they have always represented the peak in blood donation numbers since 2005. Already in 2020, the number of WBD in the baby‐boom generation decreased in comparison to previous years. This shows that these donors now begin to leave the donor pool (as predicted by our group previously in Greinacher et al. [4]), most likely due to their own age‐related health issues. Consequently, a further decline in blood donation numbers should be expected within the next 10–15 years, when many blood donors belonging to the baby‐boom generation will most likely exit the donor pool. Liberalization of the upper age limit for blood donors in Germany [15] somewhat mitigates this effect. In 2020, the age group >69 years already accounted for 1.3% of all WBD. But this will by no means compensate for the loss of WBD of younger age groups.

In previous analyses of our study until 2015, the decrease of WBD was primarily an effect of a shrinking population number in younger age groups [4, 6, 7]. Blood donor motivation remained rather stable, as indicated by only slightly decreasing blood donation rates per 1000 inhabitants in the respective age groups. In 2019, for the first time, we see a marked decline in blood donation rates per 1000 inhabitants by −13.3% compared to 2015. This decrease is especially pronounced in younger blood donors in the age groups 18–30 years. This is an extremely important observation, as it shows that the decline in blood donations in this age group is no longer solely a direct function of the age group specific population number. Rather, this reflects a decline in donor motivation and a substantial decline in first‐time donors in the younger age groups. The authors would like to emphasize that the decline in the donation rate in Mecklenburg‐Western Pomerania is by no means an actively induced reduction in WBD in response to the declining need for RBCs. On the contrary, the current WBD numbers can only be maintained with great efforts of the donor recruitment teams of all involved blood donation services.

Projections based on the actual donation rates predict a further decline of absolute WBD numbers within the next 10 years by more than 10%. Using both a population‐based projection (model A) and a behaviour‐adjusted projection (model B), we estimate that donation numbers will continue to fall, with the behaviour‐adjusted model predicting an even more pronounced decline (−12.7% model A and −15.1% model B). During the last decade, the decrease in WBD had been compensated by a parallel decrease in transfusion demand due to changes in medical practice. This has also been the case in other high‐income countries, for example, Germany [16], the United Kingdom [17] or the United States [11]. In this regard, it is important to notice that the trend of a declining RBC demand in Germany has stopped and transfusion demand numbers remain stable or even increase slightly since 2021 [16]. Jones et al. [11] report a similar development in the United States in their 2019 National blood collection and utilization survey. Already in 2019, 66% of all RBCs in Mecklenburg‐Western Pomerania were transfused to patients older than 65 years. As the baby‐boom generation now enters this age group, this will inevitably lead to an increase in transfusion demand.

Our prospective study has predefined 5 years data collection timepoints. Due to the pandemic in 2020 and 2021, we also present the data of 2019. Overall, the effects of the pandemic on absolute WBD numbers were moderate (−8.3%) paralleled by a reduced donation rate per 1000 inhabitants (−8.2%). This indicates resilience of the blood donor population during times of societal changes and crisis (at least within the study area) and underscores the importance of a long‐term, stable pool of repeat whole blood donors to prepare the health care system for crises.

Characteristics on blood donors and blood recipients including sex and age should be monitored regularly. This should ideally be done by the regulatory bodies of the individual European countries, in Germany, for example, the Paul Ehrlich‐Institute and the Robert Koch‐Institute [18].

Our findings, although derived from a German federal state, likely reflect a broader trend relevant to other high‐income countries. Similar declines in donor availability or participation rates have been reported for example in Denmark [5], the United Kingdom [17] and the United States [11], indicating that demographic ageing and reduced engagement of younger donors are not unique to Germany. The European population older than 65 years is expected to increase by 43.5% from 90.5 million in 2019 to 129.8 million people by 2050 [19], so many high‐income nations face similar demographic shifts with shrinking younger populations, ageing donor pools and changing societal attitudes toward blood donation. These factors collectively threaten the long‐term stability of blood supplies, even in healthcare systems with currently adequate resources. The data presented here may serve as an early warning for other high‐income regions, highlighting the urgent need for targeted strategies to recruit and retain young blood donors and to secure a sustainable blood supply in ageing societies.

In conclusion, transfusion safety also includes sufficient availability of blood. Monitoring numbers of blood donations per age group and transfusion demand is helpful to identify trends. In this regard, the data presented here strongly indicate a worrisome qualitative change causing a decline in the motivation to donate blood. This aggravates the effects of the demographic change. Transfusion medicine needs to counteract this, especially in the age groups 18–30 years to secure the blood supply during the next decade.

## CONFLICT OF INTEREST STATEMENT

A.G. reports grants and non‐financial support from Aspen, Boehringer Ingelheim, MSD, Bristol Myers Squibb (BMS), Paringenix, Bayer Healthcare, Gore Inc., Rovi, Sagent, Biomarin/Prosensa, personal fees from Aspen, Boehringer Ingelheim, MSD, Macopharma, BMS, Chromatec, Werfen, non‐financial support from Boehringer Ingelheim, Portola, Ergomed, GTH e.V. outside the submitted work. L.S. received a young investigator grant of the medical faculty of the Universitätsmedizin Greifswald and a Global Research Award of the American Society of Haematology, and a ‘Wiedereinstiegsförderung’ from the Else Kröner‐Fresenius‐Stiftung. L. Schönborn reports personal fees from Baxter, Viatris and GTH e.V. outside the submitted work. T.T. reports personal fees from Bristol Myers Squibb, personal fees from Bayer, Daichii Sankyo, Pfizer, Novo Nordisk, Novartis, Leo Pharma and Chugai; all of which are outside the submitted work. S.O. reports travel fees from Kite Gilead outside the submitted work. K.W., K.S., D.G., A.S. and W.H. report no conflicts of interest.

## Data Availability

The datasets generated and analyzed during this study are not publicly available due to data protection regulations and agreements with participating blood donation services. Aggregated data and statistical analysis results are available from the corresponding author upon reasonable request and subject to approval by the data providers.
